# Optimization of sand fly embryo microinjection for gene editing by CRISPR/Cas9

**DOI:** 10.1371/journal.pntd.0006769

**Published:** 2018-09-04

**Authors:** Ines Martin-Martin, Azadeh Aryan, Claudio Meneses, Zach N. Adelman, Eric Calvo

**Affiliations:** 1 Laboratory of Malaria and Vector Research, National Institute of Allergy and Infectious Diseases, National Institutes of Health, Rockville, Maryland, United States of America; 2 Department of Entomology and Fralin Life Science Institute, Virginia Tech, Blacksburg, Virginia, United States of America; 3 Department of Entomology and Agrilife Research, Texas A&M University, College Station, Texas, United States of America; Yale School of Public Health, UNITED STATES

## Abstract

**Background:**

Clustered Regularly Interspaced Short Palindromic Repeats (CRISPR)/Cas9 technology has rapidly emerged as a very effective tool for gene editing. Although great advances on gene editing in the medical entomology field have arisen, no attempts of gene editing have been reported in sand flies, the vectors of Leishmaniasis.

**Methodology/Principal findings:**

Here, we described a detailed protocol for sand fly embryo microinjection taking into consideration the sand fly life cycle, and manipulation and oviposition requirements of this non-model organism. Following our microinjection protocol, a hatching rate of injected embryos of 11.90%-14.22% was achieved, a rate consistent with other non-model organism dipterans such as mosquitoes. Essential factors for the adaptation of CRISPR/Cas9 technology to the sand fly field were addressed including the selection of a target gene and the design and production of sgRNA. An *in vitro* cleavage assay was optimized to test the activity of each sgRNA and a protocol for *Streptococcus pyogenes* Cas9 (spCas9) protein expression and purification was described. Relevant considerations for a successful gene editing in the sand fly such as specifics of embryology and double-stranded break DNA repair mechanisms were discussed.

**Conclusion and significance:**

The step-by-step methodology reported in this article will be of significant use for setting up a sand fly embryo microinjection station for the incorporation of CRISPR/Cas9 technology in the sand fly field. Gene editing strategies used in mosquitoes and other model insects have been adapted to work with sand flies, providing the tools and relevant information for adapting gene editing techniques to the vectors of Leishmaniasis. Gene editing in sand flies will provide essential information on the biology of these vectors of medical and veterinary relevance and will rise a better understanding of vector-parasite-host interactions.

## Author summary

The CRISPR/Cas9 system, based on the adaptive immune system in bacteria and archaea against viral infections, has been adapted and has rapidly emerged as a very effective genetic engineering tool in many organisms. Although great advances on gene editing in the medical entomology field have arisen, no attempts have been reported in sand flies, the vectors of *Leishmania* spp. Leishmaniasis is one of the most neglected parasitic diseases with twelve million people affected worldwide. Despite their importance as disease vectors, sand fly genetics and molecular studies are limited when compared to other insects. In this article, gene editing strategies used in mosquitoes and other model insects have been adapted to work with sand flies, providing the tools and relevant information for adapting embryo microinjection techniques to sand flies, an essential step in a successful gene editing experiment. We believe gene editing in sand flies will provide essential information of medical and veterinary relevance on the biology of these vectors, and will further a better understanding of vector-parasite-host interactions.

## Introduction

Phlebotomine sand flies (Diptera: Psychodidae) are the vectors of Leishmaniases, a group of complex parasitic vector-borne diseases that comprise diverse clinical manifestations in humans, ranging from self-healing cutaneous leishmaniasis to life-threating visceral diseases. Leishmaniases are diseases of great public health concern with an estimated incidence of 0.9–1.6 million new cases each year around the world [1]. The causative agents are several species of the genus *Leishmania* spp. (Kinetoplastida: Trypanosomatidae) which are transmitted to the vertebrate host through the bite of infected sand flies [2].

Despite their importance as disease vectors, sand fly genetics and molecular studies are limited when compared to other insects [3]. One of the main drawbacks is the lack of genome sequence information for most of the sand fly species. To date, only two genome sequencing projects are publicly available; one from the New World species *Lutzomyia longipalpis* and another from the Old World species *Phlebotomus papatasi* [4]. Several sand fly transcriptomes are available [5], mainly focused on salivary glands [6–10], sand fly-*Leishmania* interactions [11, 12] or specific tissues such as the sex pheromone gland [13]. Functional genomic studies have emerged as a potent tool to unravel the molecular mechanisms of the vector-parasite interface. Gene silencing by RNA interference (RNAi) has been widely applied in entomology for the last two decades [14]. Most recently, RNAi has also been incorporated to sand fly studies to address several questions [15–17]. However, no attempts of gene editing in sand flies have been reported in the literature.

CRISPR, the acronym for “Clustered Regularly Interspaced Short Palindromic Repeats”, along with the CRISPR associated proteins (Cas) are part of the adaptive immune system in bacteria and archaea against viral infections [18]. Recently, the CRISPR/Cas system has been adapted for genome engineering and it has rapidly emerged as an effective tool for gene editing in many organisms. Cas9 acts as an RNA-guided endonuclease that specifically recognizes and cleaves the target DNA by base-paring between single guide RNA (sgRNA) and the target sequence (protospacer), creating a double strand break (DSB) [19]. The DSBs are mostly repaired by error-prone non-homologous end joining (NHEJ) that cause gene disruption by introducing insertions or deletions, or by homology-directed repair (HDR), in which genes are replaced by recombination and a homologous sequence is required [20].

In the entomology field, the CRISPR/Cas9 system has triggered a revolution in the study of diverse arthropods including the fruit fly *Drosophila melanogaster* [21], the silkworm *Bombyx mori* (Lepidoptera) [22], the red flour beetle *Tribolium castaneum* (Coleoptera) [23] and the European honey bee *Apis mellifera* (Hymenoptera) [24] among others [25]. In the vector biology field, the CRISPR/Cas9 system has enabled studies of both Culicinae and Anophelinae mosquitoes relevant to mosquito biology, insecticide resistance and vector control strategies [26–36].

In the present paper, we describe the methodology for sand fly embryo microinjection and we discuss several essential factors for the incorporation of CRISPR/Cas9 methodology in the sand fly field. We believe that this powerful gene-editing tool will be useful for the sand fly community to better understand the sand fly biology and will help to decipher vector-parasite-host interactions.

## Methods

### Ethics statement

Public Health Service Animal Welfare Assurance #A4149-01 guidelines were followed according to the National Institute of Allergy and Infectious Diseases (NIAID) and the National Institutes of Health (NIH) Animal Office of Animal Care and Use (OACU). Sand fly maintenance was carried out according to the NIAID-NIH animal study protocol (ASP) approved by the NIH Office of Animal Care and Use Committee (OACUC), with approval ID ASP-LMVR4E.

### Selection of target gene

Sand fly genetic information is scarce when compared to the fruit fly or mosquito genomic resources. There are two sand fly genomes annotated so far: *Lu*. *longipalpis* (Lutz & Neiva, 1912) Jacobina strain, vector of visceral leishmaniasis in the New World and *Phlebotomus papatasi* Israeli strain, vector of cutaneous leishmaniasis in the Old World [4]. We encourage comparing the target gene sequence from the sand fly colony with the annotated gene in the databases (VectorBase or NCBI) before designing the sgRNA, as single nucleotide polymorphisms may be enough for changing the protospacer adjacent motif (PAM) or sgRNA recognition by Cas9. The steps followed for selection of the target gene are listed below:

1. Primer design of the target gene was based on annotated databases.
2. Genomic DNA (gDNA) from whole sand flies or specific tissues of interest was isolated by DNeasy Blood and Tissue Kit (QIAGEN) following manufacturer’s instructions.
3. PCR was performed with specific primers (0.2 μM) to amplify the gene of interest from 200 ng of gDNA with Platinum High Fidelity Taq polymerase (Invitrogene). PCR conditions were as follows: 2 min at 94 °C, 35 cycles of 15 sec, 94 °C, 30 sec at the annealing temperature specific for the primers used and an extension step of 68 °C for 1 min/kb.
4. PCR product was purified (SpinPrep PCR Clean-up Kit, Millipore) and an aliquot of 20–40 ng/μl was sent for sequencing.
5. Electropherograms were manually inspected and aligned with the annotated sequences using SeqMan Pro (DNASTAR, Lasergene 12).

### sgRNA design

Once the target DNA sequence region was verified, specific sgRNA were designed using CHOPCHOP v2 software [37] (http://chopchop.cbu.uib.no/index.php#) which contains the *Lu*. *longipalpis* Jacobina strain LlonJ1 genome [4]. Default parameters for designing sgRNA with CHOPCHOP software v2 were set: off-target with up to 3 mismatches in protospacer [38], with an efficiency score based on Xu *et al*. [39] and self-complementary according to Thyme *et al*. [40]. The chosen sgRNA sequences need to be incorporated into forward primers along with the T7-promoter region and a sequence complementary to a common reverse primer (Scaffold-R, Table 1), part that would bind the Cas9 protein.

**Table 1:** sgRNA targeting *LuloYLW* gene.

sgRNA	5’- T7-promoter Gene-specific (20mer) Scaffold-F (CRISPR) -3’	Genomic location	PAM	Strand	GC (%)	Self-complementarity	Off-targets	Efficiency
LuloYLW_1	**GAAATTAATACGACTCACTATAGG**ATATGGGCAGGGATTCCTAGGTTTTAGAGCTAGAAATAGC	Scaffold614:35631	TGG	-	52	0	0	0.57
LuloYLW_2	**GAAATTAATACGACTCACTATAGG**GACATTGAGAGCATATGGGCGTTTTAGAGCTAGAAATAGC	Scaffold614:35643	AGG	-	52	0	0	0.62
LuloYLW_3	**GAAATTAATACGACTCACTATAGG**TCATATTGGTGTCCTCAGGTGTTTTAGAGCTAGAAATAGC	Scaffold614:35707	CGG	-	52	0	0	0.49
LuloYLW_4	**GAAATTAATACGACTCACTATAGG**GCGCTACGTTCTCCGACCTGGTTTTAGAGCTAGAAATAGC	Scaffold614:35695	AGG	+	65	0	0	0.57
LuloYLW_5	**GAAATTAATACGACTCACTATAGG**CTGATGTCTGCGGTAGGCTTGTTTTAGAGCTAGAAATAGC	Scaffold614:35574	TGG	+	57	0	0	0.49
LuloYLW_6	**GAAATTAATACGACTCACTATAGG**CGCATCAAGGCTGATGTCTGGTTTTAGAGCTAGAAATAGC	Scaffold614:35564	CGG	+	61	1	0	0.46
Scaffold-R	AAAAGCACCGACTCGGTGCCACTTTTTCAAGTTGATAACGGACTAGCCTTATTTTAACTTGCTATTTCTAGCTCTAAAAC							

### sgRNA production

DNA templates for each sgRNA were produced by PCR with specific primers followed by *in vitro* transcription. The steps are listed below:

1. PCR amplification of overlapping primers was performed with Platinum PCR SuperMix following manufacturer’s instructions (Invitrogen).
2. Purification of DNA was carried out with SpinPrep PCR Clean-up Kit (Millipore) and used as template for transcription reaction (500 ng of starting material per reaction) with the MEGAscript T7 Transcription Kit (Ambion).
3. Transcription reactions were run for 20 h followed by DNase*I* treatment (20 min at 37 °C).
4. RNA concentration was determined by Nanodrop ND-1000 spectrophotometer.
5. sgRNAs were resuspended with DEPC-H_2_0 and adjusted to 2500 ng/μl and stored at -80 °C in 10 μl single use aliquots.

### *Streptococcus pyogenes* Cas9 production

For Cas9 mRNA production, pRGEN-Cas9-CMV plasmid (PNA Bio) was linearized by *Xho*I over-night digestion at 37 °C and used as a template for transcription reaction following mMessage mMachine T7 Ultra kit instructions (Ambion).

Cas9 protein expression and purification was performed as described by Zuris *et al*. [41] with several modifications:

1. *Escherichia coli* BL21 STAR (D3)-competent cells (Invitrogen) were transformed with pET-NLS-Cas9-6xHis plasmid (Addgene 62934).
2. Transformed bacteria were grown in 100 ml of Luria-Bertani (LB) broth in the presence of 100 μg/ml ampicillin overnight at 250 rpm, 37 °C.
3. The following day, cells were 1:100 diluted in fresh LB broth with antibiotic (1-liter culture) and incubated until culture reached OD_600_ of 0.6–0.7.
4. Protein expression was induced with 1 mM isopropyl β-D-1- thiogalactopyranoside for 16 h at 22 °C.
5. Bacterial cells were collected by centrifugation at 6,000 rpm for 15 min at 20 °C. Cells were resuspended in 10 mM Tris-HCl, 500 mM NaCl, pH 8.0 and lysed by sonication (3 pulses of 30 sec each at 50W, kept on ice for 30 sec between pulses).
6. Cell lysate was cleared by 2 step-centrifugation (first centrifugation at 10,000 rpm for 10 min and a ultracentrifugation of the cloudy supernatant at 40,000 rpm for 30 min at 4 °C, Beckman Coulter).
7. Recombinant Cas9 protein was purified from the soluble cell lysate by affinity and cation exchange chromatography:
  - For affinity chromatography, cell lysate was passed through a 0.1 M Nickel-charged 5 ml HiTrap Chelating HP (GE Healthcare Life Science, Piscataway, NJ) and the protein was eluted by creating a gradient of Imidazole (0–500 mM) that released the binding of the Nickel and the His-tag.
  - Chromatography fractions were checked on a 4–12% NuPage gel (Life Technologies) and proteins were visualized by Coomassie stain.
  - Fractions that contain the recombinant Cas9 were combined and NaCl was removed by dialysis with 25 mM 2-(N-Morpholino) ethanesulfonic acid (MES), pH 6.0 at 4 °C overnight using dialysis cassettes (Thermo Scientific, MWCO 10 kDa).
  - The protein was further purified by cation exchange chromatography on a MonoS 5/50 GL column (GE Healthcare Life Science, Piscataway, NJ). The protein was eluted by increasing ion strength (0–1000 mM NaCl).
  - Chromatography fractions were visualized on a gel and proper fractions were combined.
  - Protein was dialyzed with 25 mM Tris-HCl, 150 mM NaCl, pH 7.3 overnight using dialysis cassettes (Thermo Scientific, MWCO 10 kDa).
  - The concentration was determined based on the assumption that 1 mg ml^-1^ has an absorbance at 280 nm of 0.76, according to the molecular extinction coefficient (120,700 M^-1^ cm^-1^).

All protein purification experiments were carried out using an AKTA purifier system (GE Healthcare Life Science, Piscataway, NJ). Home-made Cas9 protein activity was tested by comparison with commercial recombinant Cas9 protein, that was purchased from PNABio (CP02). If home-made Cas9 protein expressed in bacteria is going to be included in the injection mix, endotoxin levels should be monitored to ensure no bacteria lipopolysaccharide is microinjected into the embryo.

### *In vitro* cleavage assay

To test the ability of each sgRNA to cut the target DNA *in vitro*, we checked the integrity of the target DNA region after incubation with the sgRNA in the presence of Cas9 protein in an *in vitro* cleavage assay.

1. The target gene was amplified from gDNA or cDNA (assuming target region was within an exon) from *Lu*. *longipalpis* using Platinum DNA polymerase (Invitrogen) with 0.2 μM of specific primers. The PCR product was purified (SpinPrep PCR Clean-up Kit, Millipore) and used as template for the *in vitro* cleavage assay.
2. Cas9 protein (400 ng; 3.8 μM) was pre-incubated with each individual sgRNA (30 ng; 3.2 μM) for 10 min at 37 °C to ensure proper loading of the sgRNA.
3. Target DNA (200 ng) was mixed to pre-loaded Cas9 protein and reactions were incubated at 37 °C in the presence of 1X Bovine Serum Albumin and 1X NEB3 buffer (New England Biolabs) in a total volume of 20 μl.
4. After 1 h and 15 min incubation period, Cas9 protein was inactivated at 65 °C for 10 min.
5. As controls, template DNA were incubated with individual sgRNA in the absence of Cas9 protein. In addition, target DNA with and without Cas9 protein were included.
6. Samples and controls were run in a 0.5 μg/ml ethidium bromide 2.2% agarose gel and the loading buffer (Thermo Fisher Scientific) was supplemented with 0.1% SDS.
7. Tris-acetate-EDTA (KD Medical) was used as a running buffer for DNA electrophoresis and bands were visualized and scanned under UV light.

### Injection mix

Initially, injection mixes consisted of 600 ng/μl Cas9 mRNA and a mixture of 100 ng/μl of each sgRNA in nuclease free water. Each injection mix was freshly prepared on the day of the injection, centrifuged at 13,000 rpm at 4 °C and kept on ice during microinjections.

As an alternative to mRNA, Cas9 protein can be included in the injection mix. A final concentration of 330 ng/μl Cas9 with 100 ng/μl of each sgRNA would be in the equimolar range. Tubes containing Cas9 protein should be independently loaded with the individual sgRNAs for 10 min at 37 °C to avoid Cas9 binding sgRNA with different affinities potentially resulting in different degrees of sgRNA loading.

### Pulling needles

**Fig 1:**
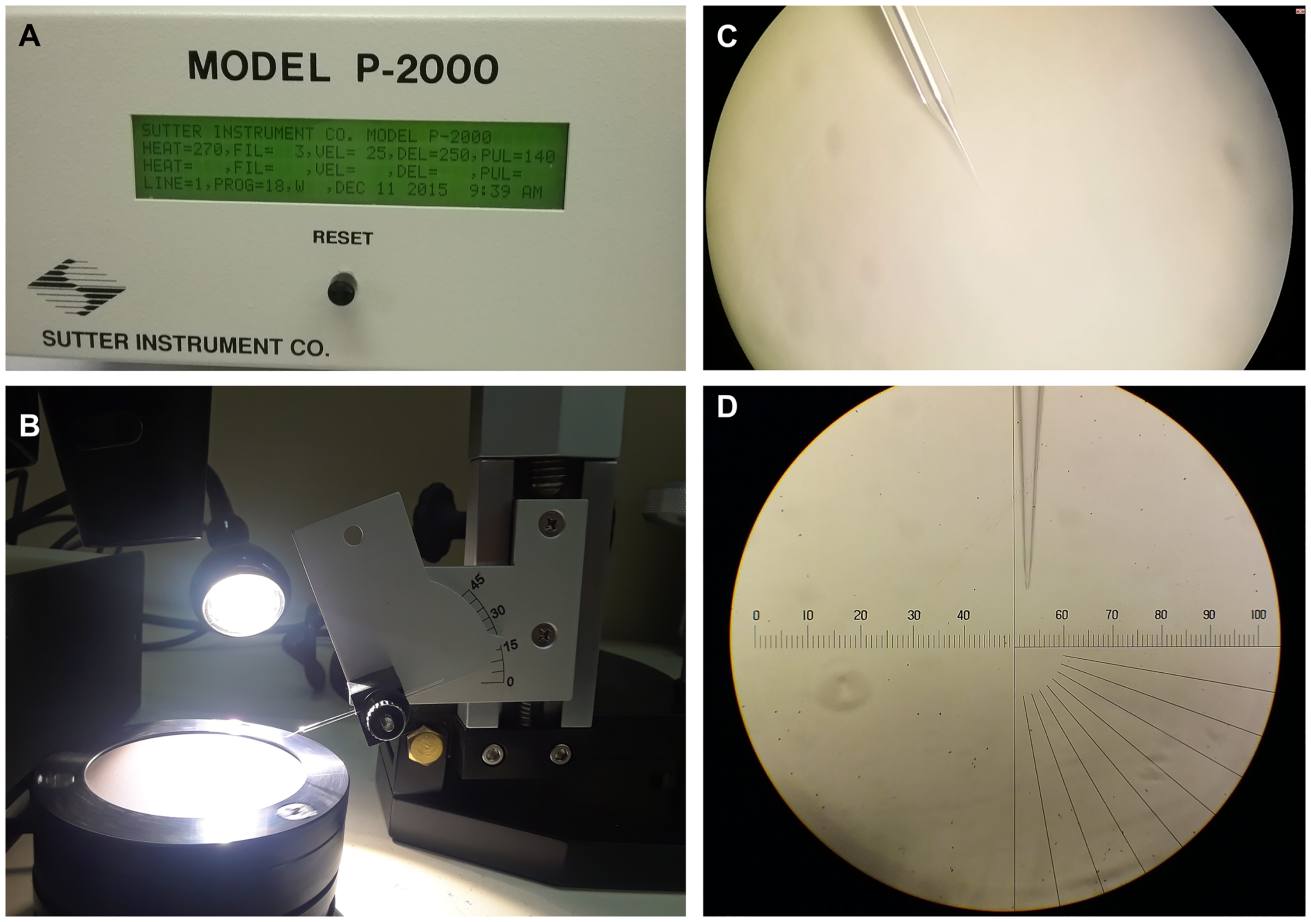
Needle preparation. **(A)** Needles were pulled using a Sutter P-2000 micropipette puller. **(B) (C)** Pulled needles were beveled to an angle of 20° using a Sutter BV-10 Microelectrode Beveller, 104D fine abrasive plate. **(D)** Needles were inspected under a microscope to ensure the bore is not greater than 1 μm. NOTE: If there is no availability of a needle beveller, needles can be opened by breaking the bore towards a glass slide under the microscope (always verify the opening is smaller than 1 μm).

Borosilicate glass capillaries needles (#1B100-4, Kwik-Fil) were laser-pulled using a Sutter P-2000 micropipette puller (Novato, CA, USA, Fig 1A) and parameters were set as follows: Heat = 270, FIL = 3, VEL = 25, DEL = 250, PUL = 140. Needles were beveled to an angle of 20° (Sutter BV-10 Microelectrode Beveller, 104D fine abrasive plate, Fig 1B and 1C). All needles were inspected under a microscope (Fig 1D) and the ones with a bore greater than 1 μm were discarded to avoid embryo damage.

### Sand fly embryo microinjection

*Lutzomyia longipalpis* Jacobina strain was reared following standard conditions at the Laboratory of Malaria and Vector Research (LMVR), National Institutes of Health (NIH). The embryo microinjection protocols as described by Aryan *et al*. [42] were followed and adapted to sand fly work.

**Fig 2:**
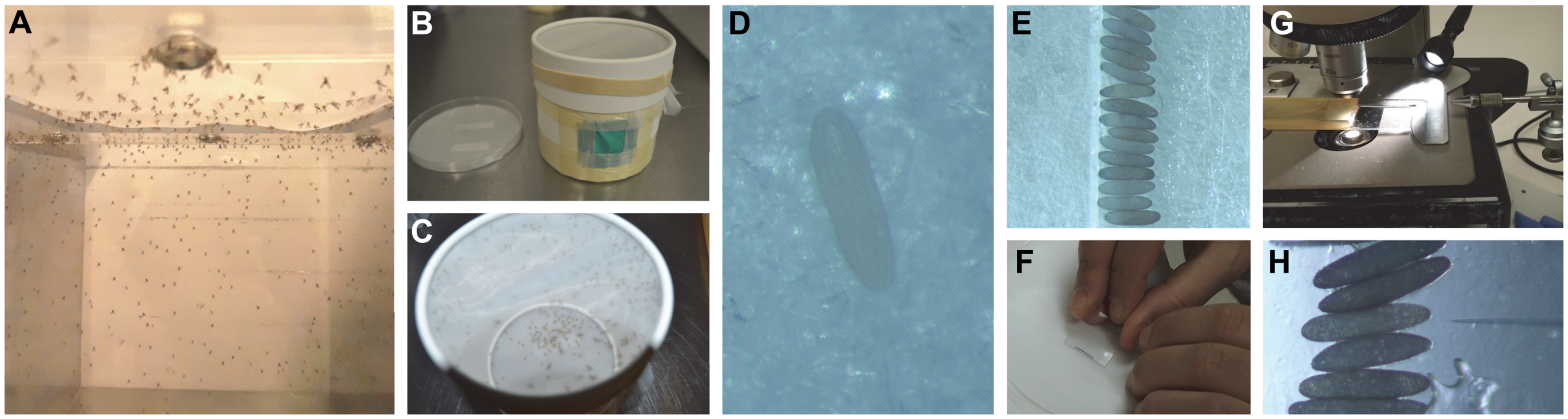
Layout of sand fly embryo microinjection. **(A)** Blood fed females were maintained in adult cages for 5–7 days. **(B, C)** Gravid sand fly females were transferred to paper cups with a humid filter paper on the bottom to promote oviposition. Sand flies were allowed to lay eggs in the dark for 1 h and recently laid eggs **(D)** were collected. **(E)** Alignment of embryos was performed on humid filter paper under a dissecting microscope. **(F)** Embryos were transferred from the filter paper to a cover slip with double-sided tape and covered with halocarbon oil to prevent desiccation. **(G)** Cover slip containing the embryos was placed on the microscope stage and **(H)** the borosilicate needle backfilled with the injection mixture was adjusted towards the posterior end of the embryo to start microinjections.

1. *Lu*. *longipalpis* blood-fed 4 to 6-day-old females were held for 5–7 days in adult cages to avoid non-synchronized egg laying (Fig 2A).
2. On the day of microinjection, gravid sand flies were transferred to manually prepared card box cages with humid hardened filter paper on the bottom (grade 50, Whatman, Fig 2B and 2C).
3. Sand flies were allowed to lay eggs in the dark for 1 h at 27 °C and freshly laid, non-melanized eggs were collected with a fine brush (Fig 2D).
4. Embryos were aligned towards a humid filter paper (grade 50, Whatman) and oriented in the same anterior-posterior direction to allow injection of material into the posterior pole. Aligned embryos were desiccated for a few seconds by drying out the filter paper before being transferred to a coverslip with double-sided adhesive tape along the edge. Embryos were immediately covered with halocarbon oil 27 (Sigma, St. Louise, MO) to prevent over-desiccation (Fig 2E and 2F).
5. The injection mix was loaded into the needle and 1 h 30 min to 3 h old *Lu*. *longipalpis* embryos were microinjected in the posterior pole using a Femtoject 4i microinjector (Eppendorf) and a Leica micromanipulator (Fig 2G and 2H).
6. After injection, halocarbon oil was removed with distilled water and injected embryos were transferred with the help of fine tweezers (Dumont #5 Inox 11 cm) or a fine brush to a small plastic beaker with humid filter paper. Embryos were kept at 27 °C for 2 days before being transferred to plaster of Paris larval pots. ([43], https://www.vectorbase.org/content/cd-sand-fly-fellas-sand-fly-rearing-guide).

## Results and discussion

Although gene editing has become a widespread practice in all fields of science, including medical entomology [44–46], genome editing based mutagenesis has not yet been documented in the sand fly model. In this article, we describe a detailed protocol to perform sand fly embryo microinjection, an essential step for CRISPR/Cas9 gene editing experiments.

**Fig 3:**
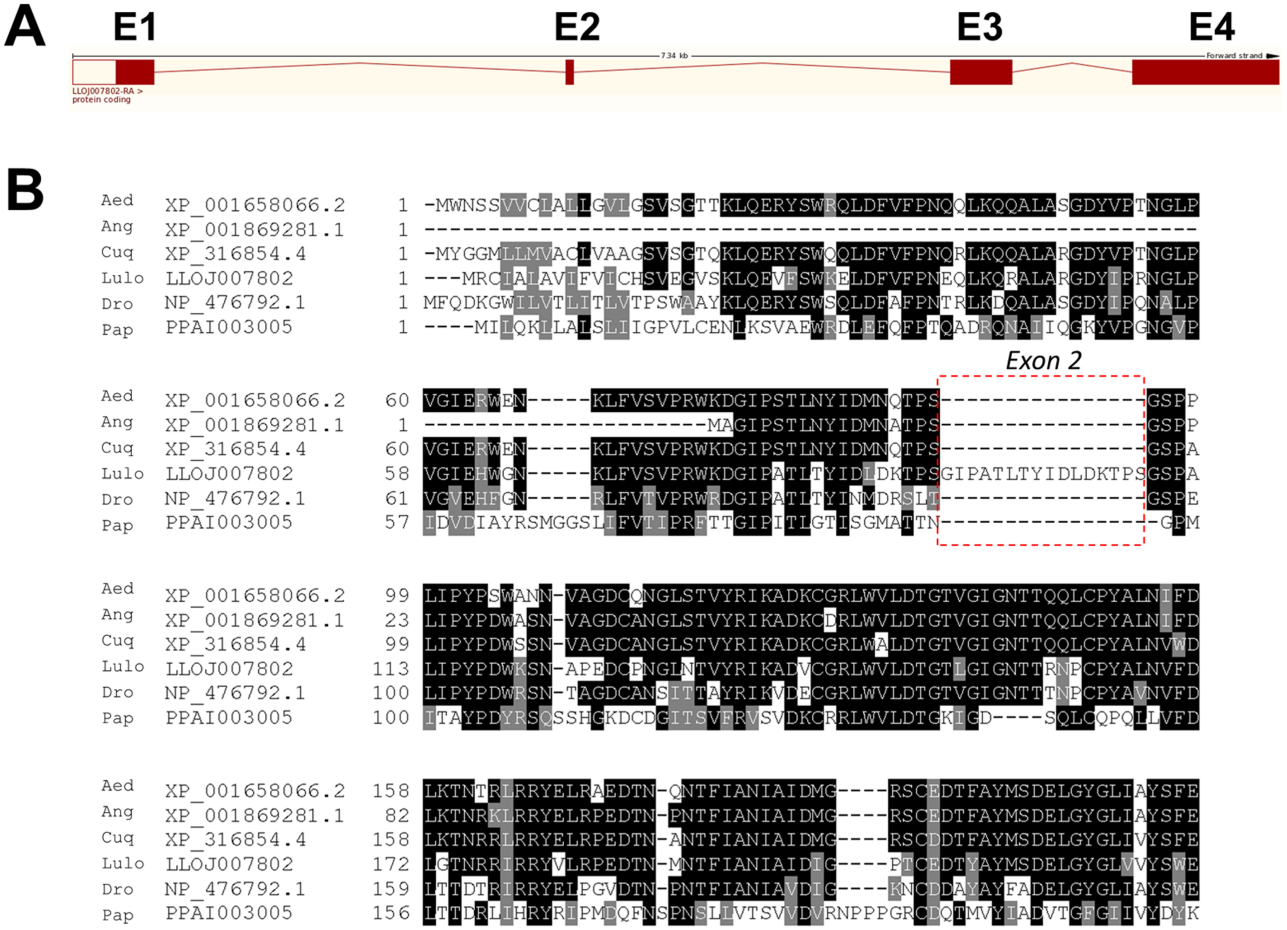
Detail on *Lutzomyia longipalpis* yellow gene intronic-exonic information and protein alignment. **(A)** Intron-exon organization of yellow gene from *Lu*. *longipalpis* E1, E2, E3 and E4 indicates the different hypothetical exons according to the VectorBase database LLOJ007802. **(B)** Multiple sequence alignment of yellow protein from sand flies *Lu*. *longipalpis* (Lulo) and *P*. *papatasi* (Pap), and other related species: *Drosophila melanogaster* (Dro), *Ae*. *aegypti* (Aed), *An*. *gambiae* (Ang) and *Culex quinquefasciatus* (Cuq). Accession numbers are indicated in the sequence name. Sequence correspondent to hypothetical exon 2 from LLOJ007802 is higlighted within a dotted box. Sequences without signal peptide were aligned with ClustalW and refined using Boxshade server, and the percent identity or similarity for shading was set at 80%. Black background shading represents identical amino acids, grey shading designates similar amino acids while white shading indicates no similarity.

The *Yellow* gene (LuloYLW), responsible for pigmentation of the sand fly body, was chosen as a target gene for the sgRNA production and embryo microinjection protocol. The Drosophila *yellow* ortholog in *Lu*. *longipalpis* was identified through tblastx analysis as LLOJ007802 (E-value: 4e-168), located in scaffold614: 30,092–37,432 in the forward strand. According to the VectorBase (VB) database, its transcript consists of 4 exons (Fig 3A) and codify a protein of 512 amino acids. To confirm these results, LLOJ007802 was sequenced using gDNA of 4 individual sand flies (2 females and 2 males). Although some single nucleotide polymorphisms were found between the LLOJ007802 transcript annotated sequence and our *Lu*. *longipalpis* sand flies, the overall similarity was maintained. However, we found that the LLOJ007802 gene consists of only 3 exons and not 4, as annotated in the VB database. The second exon designated in the VB database (5’-GAATTCCCGCCACATTGACGTACATTGATCTCGACAAGACACCATCAG-3’) is a repetition of the beginning of the third exon. Alignment with amino acid sequences from other related yellow proteins confirmed the absence of the exon 2 (Fig 3B).

**Fig 4:**
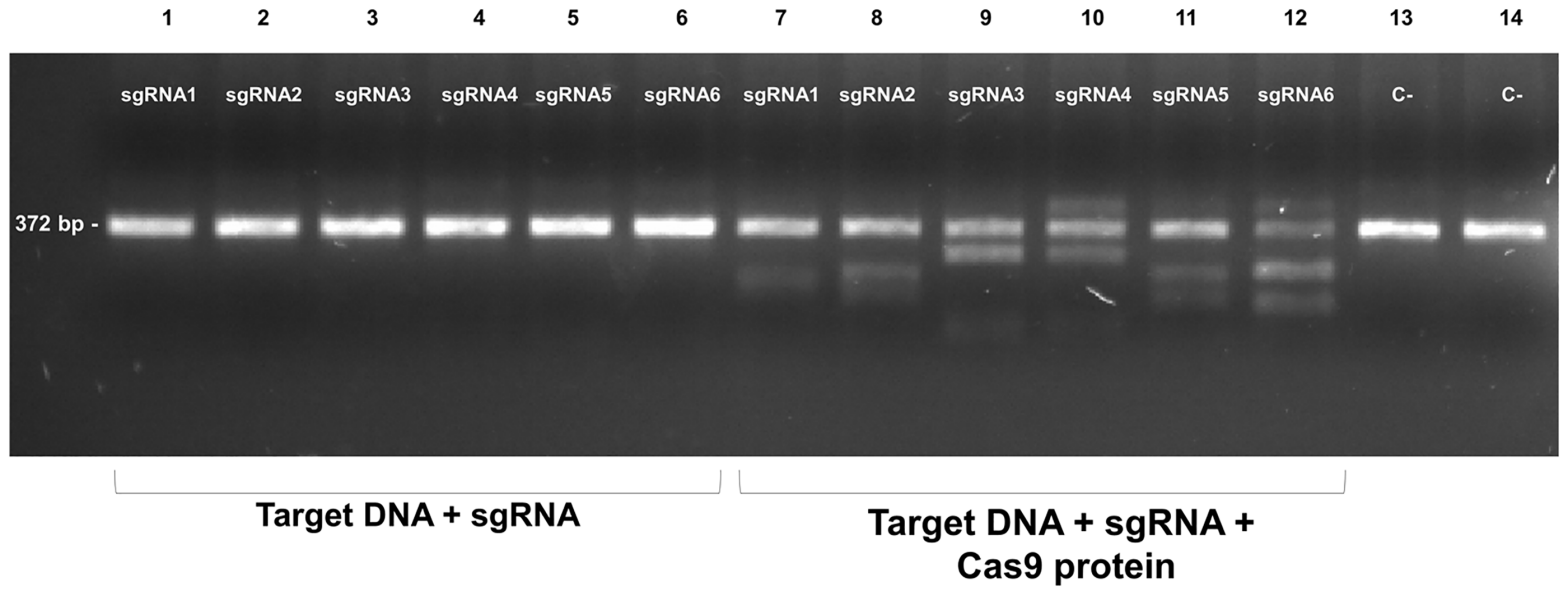
All six sgRNA produce Cas9-driven cleavage *in vitro*. 200 ng of the PCR product of the LuloYLW gene exon 3 were incubated with each individual sgRNA (3.2 μM) in the absence (lanes 1–6) or presence of 3.8 μM Cas9 protein (lanes 7–12). DNA bands size corresponds to the expected size according to each cleavage site (sgRNA1 = 218 bp, 154 bp; sgRNA2 = 230 bp, 142 bp; sgRNA3 = 294 bp, 78 bp; sgRNA4 = 279 bp, 93; sgRNA5 = 158 bp, 214 bp and sgRNA6 = 148 bp, 224 bp). As negative controls, 200 ng of PCR product alone (lane 13) or in combination with Cas9 protein (lane 14) were included. All samples were run on 2.2% agarose gels and visualized under UV light.

The LuloYLW gene was inspected in search of PAM sequences for Cas9 endonuclease (NGG) with the help of CHOPCHOP software. Six sgRNA of 20 nucleotides length were designed next to PAM sequences in the exon 3, where the major royal jelly protein domain (pfam03022) is located (Table 1). Three sgRNA in each DNA strand were chosen and both self-complementary and off-targets were avoided. To generate the sgRNA, overlapping forward specific and a complementary common reverse primer were amplified. Purified PCR products served as templates for transcription to obtain the sgRNA. Once purified, sgRNA were kept individually in 2500 ng/μl aliquots at -80 °C until used. To validate the sgRNA, we tested the ability of each sgRNA to cut the target DNA *in vitro*. The target DNA region (exon 3) was amplified from cDNA of 10 female *Lu*. *longipalpis* using Platinum DNA polymerase (Invitrogen) with 0.2 μM of specific primers (LuloYLW-E3-F: 5’-GAATTCCCGCCACATTGACG-3’ and LuloYLW-E3-R: 5’-CCAATTCGTCGGACATATAAGC-3’). Visualization of the integrity of the target DNA showed that all 6 sgRNA tested were able to drive Cas9 protein to cleave the target DNA resulting in fragments that matched the expected size, according to each cleavage site (Fig 4).

**Fig 5:**
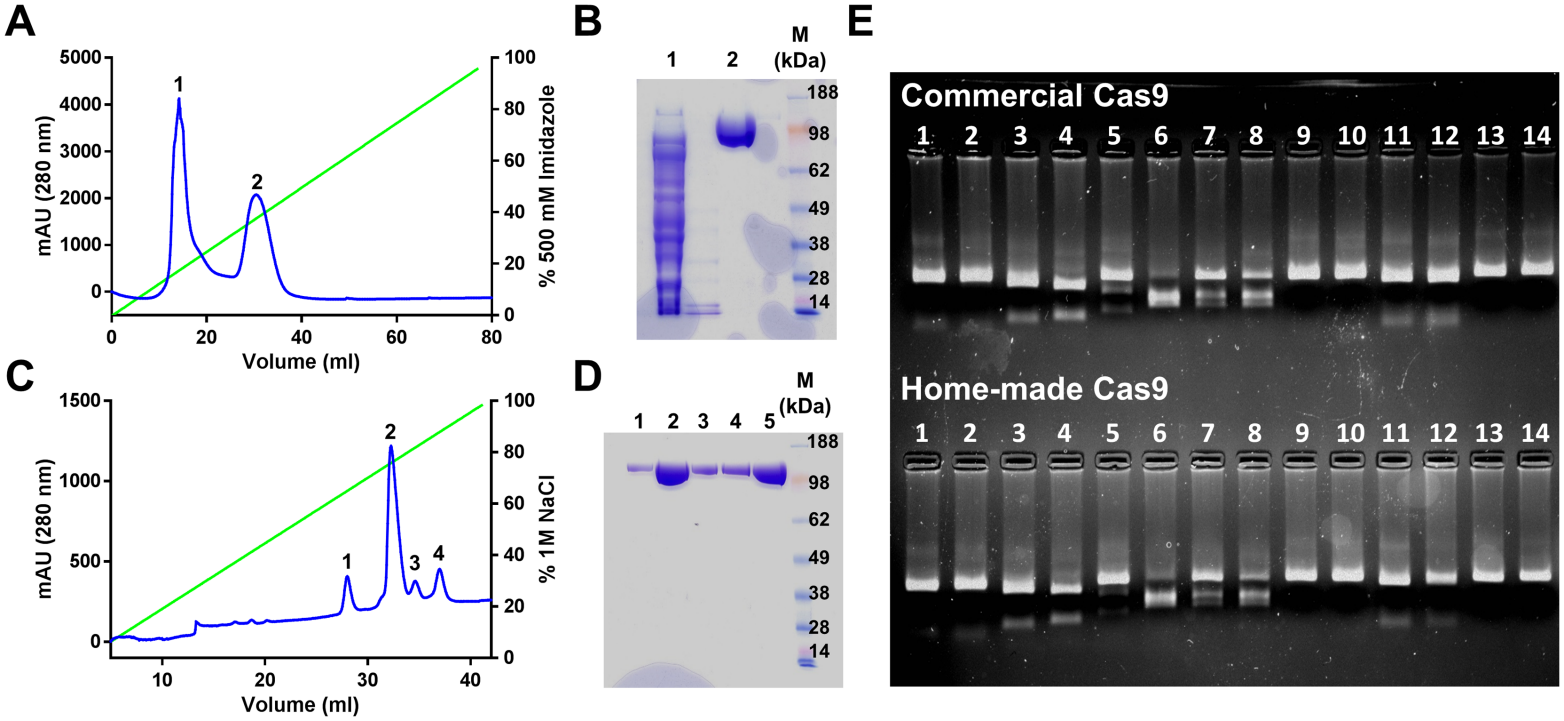
Purification of Cas9 recombinant protein. **(A)** Purification of Cas9 by affinity chromatography using a Nickel-charged HiTrap Chelating HP. Gradient of Imidazole is indicated by the green line. **(B)** Coomassie-stained gel electrophoresis of peaks 1 and 2 after affinity chromatography (26 μl of each fraction) shows that peak 2 corresponds to Cas9. M: SeeBlue Plus2 Pre-Stained Protein Standard (Life Technologies). **(C)** Purification of Cas9 by cation-exchange chromatography using a MonoS 5/50 GL column. Gradient of NaCl is indicated by the green line. **(D)** Coomassie-stained gel electrophoresis of fractions correspondent to different peaks after cation-exchange chromatography (26 μl of each fraction, peaks 1–4: lanes 1–4). All peaks showed a band of the correct Cas9 molecular weight. Only the majoritarian peak (#2) was collected. Lane 5 corresponds to purified Cas9 after dialysis. M: SeeBlue Plus2 Pre-Stained Protein Standard (Life Technologies). **(E)** Purification of recombinant Cas9 produced in our laboratory showed endonuclease activity comparable to a commercial Cas9 (PNABio). 200 ng of PCR product of target gene was incubated with each individual sgRNA (lanes 1–12; 3.2 μM) in the presence of 3.8 μM Cas9 protein either purchased from PNABio (upper gel) or obtained in our laboratory (lower gel). As negative controls, 200 ng of PCR product alone (lane 13) or in combination with Cas9 protein (lane 14) were included. All samples were run on 2.2% agarose gels and visualized under UV light.

Cas9 recombinant protein was successfully expressed and purified in our laboratory starting from the initial cloning of the commercial plasmid Addgene 62934 (Fig 5). A yield of 0.7 mg of purified protein per liter of culture was achieved with this protocol. The recombinant Cas9 protein run on a gel at the expected molecular weight and its endonuclease activity was demonstrated as shown in a side by side comparison in an *in vitro* cleavage assay with the *in house* recombinant protein and a commercial one (PNABio) using the same sgRNAs (Fig 5).

**Fig 6:**
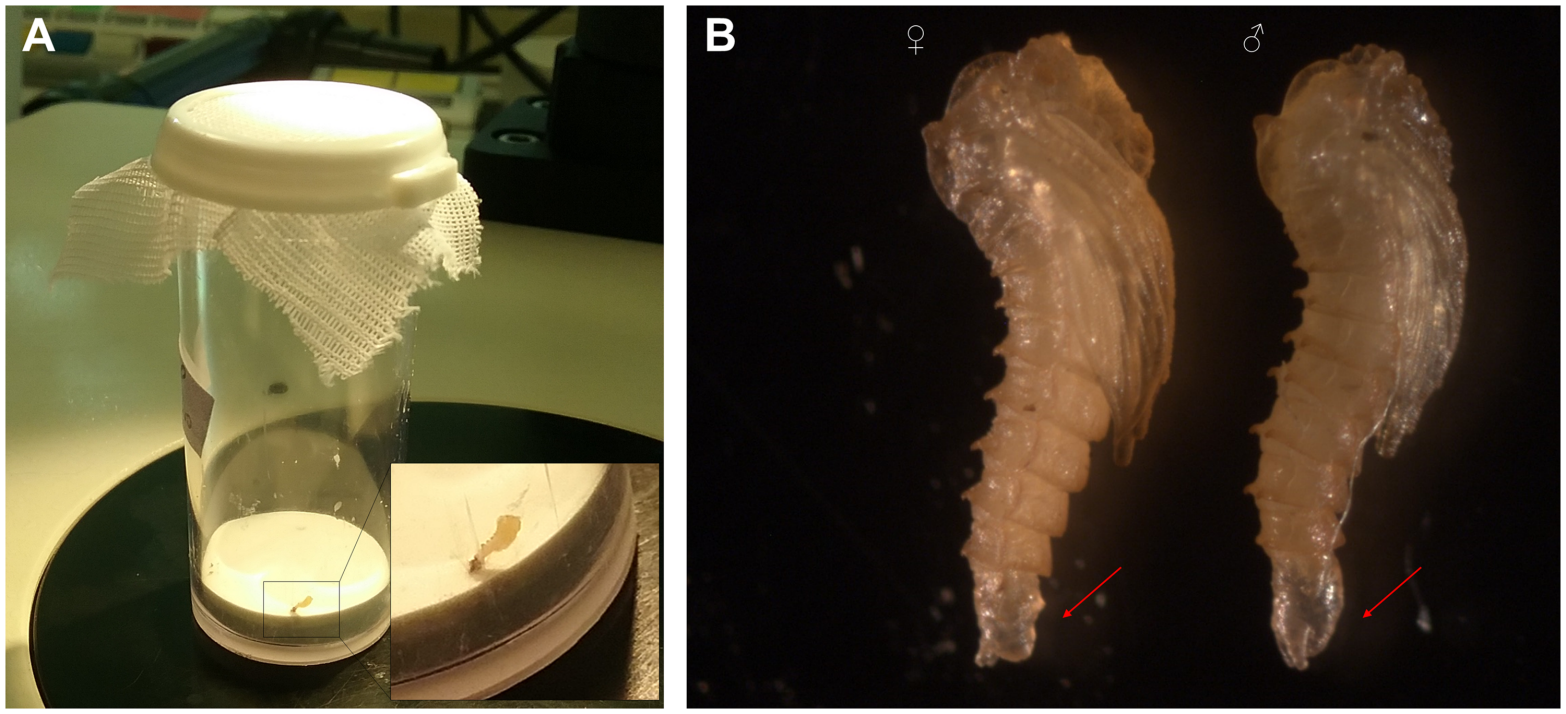
Details of G_0_ sand fly individuals and pupae sexing. **(A)** G_0_ pre-pupae were separated into individual tubes with humid plaster of Paris on the bottom to allow them to pupate. Inset image shows a magnification of a sand fly pupa in the tube. **(B)** Sand fly pupae were sexed according to the shape of the last pupal segment, which in males is larger and more rounded than in female pupa (differences indicated by red arrows).

Three sets of microinjections were carried out. A total of 775 embryos were injected with the mixture of sgRNAs and Cas9 mRNA (84, 269 and 422 for each set of injection). After microinjection, halocarbon oil was washed off and injected embryos were deposited into a beaker with humid filter paper on the bottom. Two days after microinjection, embryos were transferred to a larva pot with humid plaster of Paris base. Ten, thirty-eight and sixty embryos respectively, hatched resulting in hatching rates of 11.90%, 14.13% and 14.22%, values slightly lower to other non-model insects, such as mosquitoes [47, 48]. In this specific setting, hatching rates of non-injected wild type embryos were 64.7%. From the third set of microinjections, larvae were followed. These 60 larvae were separated at prepupae stage in individual polypropylene vials (height 5.4 cm, diameter 2.2 cm) with plaster of Paris on the bottom (Fig 6A) to maintain proper humidity. 42 G_0_ larvae survived and were sexed (20 males and 22 female pupae). It is important to note that germ-line mutagenesis experiments require virgin female adults, which can be easily obtained by sexing pupae according to differences in the last pupal segment as shown in Fig 6B.

Nuclease activity of *Streptococcus pyogenes* Cas9 (SpCas9) can be triggered when there is imperfect complementarity between the sgRNA and a genomic site leading to genomic off-target mutagenesis. Lately, engineered Cas9 with improved specificity, such as eSpCas9, SpCas9-HF or HypaCas9, have arisen providing a more robust on-target cleavage [49–51]. Although we used SpCas9 in our experiments, it would be beneficial for future studies to substitute the SpCas9 with any of their improved versions.

**Fig 7:**
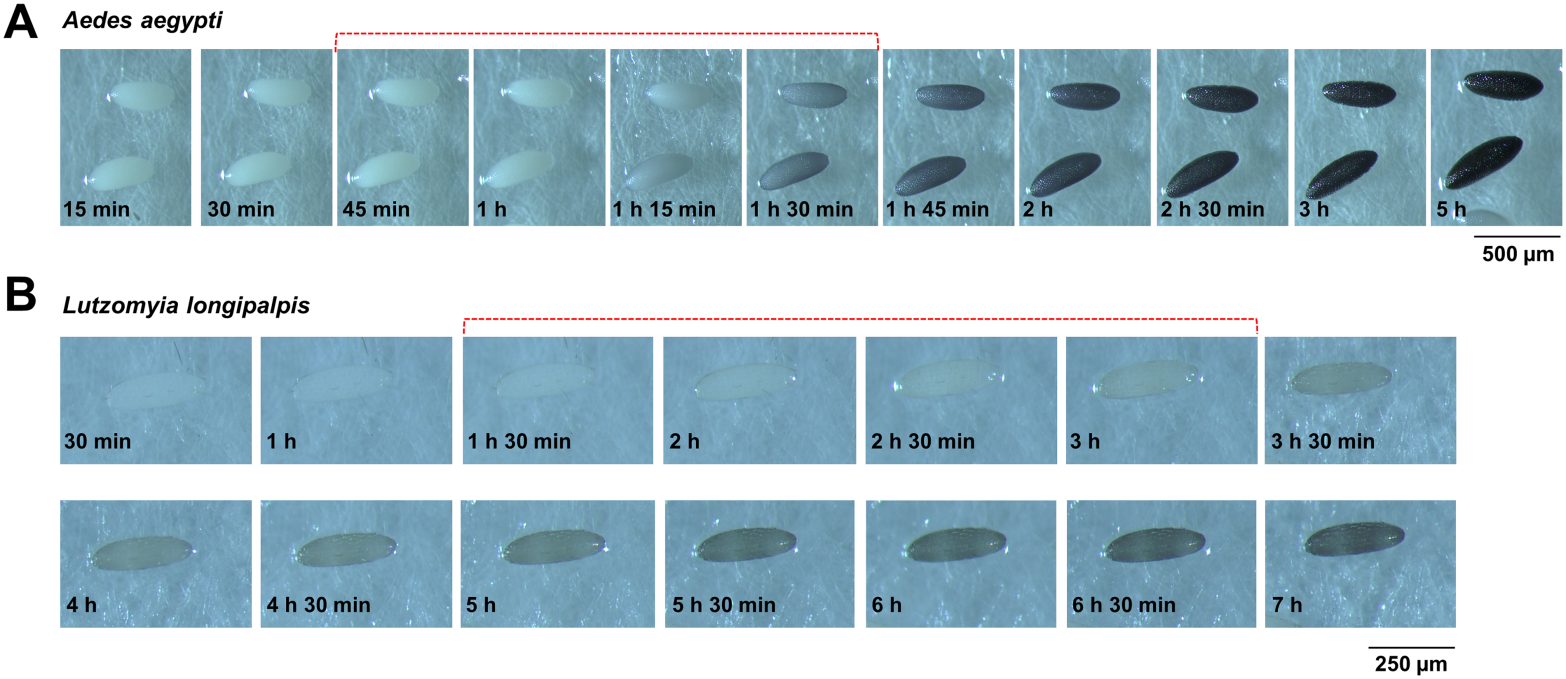
Melanization of embryos over time. Preferred melanization stages for injection are indicated with dotted brackets. **(A)** *Aedes aegypti* embryos are best injected between 45 min and 1 h 30 min, when they are getting darker, so their chorion is hardened enough to protect the embryo from bursting but not too hard that they would break the needle. Scale bar = 500 μm. *Aedes aegypti* (Liverpool strain, LMVR, NIH) gravid female mosquitoes were transferred to a 50-ml tube (Falcon, Fisher Scientific) with a humid filter paper on the bottom to promote egg laying. **(B)** *Lutzomyia longipalpis* takes longer to start melanizing. The time frame for optimal *Lu*. *longipalpis* embryo microinjections is between 1 h 30 min and 3 h after oviposition. Scale bar = 250 μm. For visualization of the melanization process, both *Ae*. *aegypti* and *Lu*. *longipalpis* were allowed to lay eggs for five min.

Having a successful microinjection for germ line transformation depends mainly on two factors. The first factor is the melanization process of the egg. Upon oviposition, sand fly embryos, like other dipterans, start to melanize and harden. Injection of freshly laid embryos will result excessive damage resulting in the death of the embryos since the chorion is not sufficiently hardened. On the other hand, when they are too melanized, the needle will be unable to penetrate the hardened chorion. In our observations, the proper time for injection of sand flies regarding melanization was between 1 h 30 min and 3 h after egg laying. In that period of time *Lu*. *longipalpis* embryos have acquired the appropriate degree of melanization for microinjections that simultaneously involve hardening of the chorion. Fig 7 illustrates the melanization process over time in *Aedes aegypti* and *Lu*. *longipalpis*. The time window is greater than with mosquitoes which need to be injected within minutes after aligning [42, 52, 53].

The second factor that determines the success of the germ line gene editing is the localization of the injection mix within the embryo and peculiarities of embryology (ie, location for developing pole cells/ germ cells). The injected material needs to be delivered at pre-blastoderm stage when the polar cells are forming and before cellularization occurs. Information on sand fly embryology is scarce. The few reports describing the embryology of *P*. *papatasi* indicate that pole cells formation occurs in the posterior end of the egg, just beneath the vitelline membrane by 36 h after oviposition [54]. Pole cells formation in sand flies takes place much later than in *D*. *melanogaster* or mosquitoes which occurs around 1 h 30 min after egg laying [42, 55]. This is concordant with the embryonic development time (from oviposition until hatching) in the different insect species. It takes 1 day for fruit fly embryos to hatch [55], 2–3 days for mosquito embryos [56] and more than a week for sand fly embryos [43]. Depending on the sand fly species, development time varies (9 days for *P*. *papatasi* [54], 8 days for *Lu*. *longipalpis* Jacobina strain [57]). Therefore, the time window for microinjection to target the polar cells in sand flies is substantially wider. Hence, what determines the injection time window is the hardness of the cuticle as discussed above.

The CRISPR/Cas9 system uses sgRNA to specifically cleave the DNA resulting in double-stranded break (DSB) in the genome [21]. DSB are repaired by classical non-homologous end joining (C-NHEJ) or homologous directed repair (HDR). Although both repair mechanisms are competitive, C-NHEJ is strongly preferred over HDR in *Ae*. *aegypti* [30]. Other repair mechanisms such as alternative non-homologous end joining (A-NHEJ) or single-strand annealing (SSA) can also play a role [58]. The NHEJ pathway is an error-prone mechanism that cause gene disruption by introducing insertions or deletions, whereas for HDR, genes are replaced by recombination and a homologous sequence is required. Unravelling the DNA repair mechanisms is essential to understanding gene editing outcomes. A lack of information on DNA repair pathways in sand flies makes it difficult to estimate the potential knock-out or knock-in outcomes. To gain information on DSB mechanisms in sand flies, a bioinformatic search for orthologs involved in NHEJ, HDR or SSA pathways was carried out (Table 2). Most of the known genes involved in DSB repair in *Anopheles gambiae* or *D*. *melanogaster* were also identified in *Lu*. *longipalpis* indicating that all DSB repair mechanisms are potentially feasible.

**Table 2:** Orthologs of DNA break repair genes in *Lutzomyia longipalpis*.

Gene	C-NHEJ	A-NHEJ	HDR	SSA	*An*. *gambiae*	*D*. *melanogaster*	*Lu*. *longipalpis*	Location	Strand	Search method	*e-value*
Ku70	x				AGAP002690	FBgn0011774	LLOJ003544	Scaffold21: 254,199–263,494	Reverse	tblastn to *An*. *gambiae* gene	5.00E-135
Ku80	x				AGAP009910	FBgn0041627	LLOJ009466	Scaffold876: 4,739–6,975	Reverse	tblastn to *An*. *gambiae* gene	2.00E-134
DNA-PKcs	x				AGAP003967	Absent	LLOJ003970	Scaffold2350: 929–24,122	Forward	tblastn to *An*. *gambiae* gene	0
Ligase 4	x				AGAP000623	FBgn0030506	LLOJ008477	Scaffold71: 184,650–188,282	Reverse	tblastn to *An*. *gambiae* gene	4.00E-15
Artemis	x				AGAP000597	Not identified	LLOJ009728	Scaffold92: 6,692–12,025	Reverse	tblastn to *An*. *gambiae* gene	8.00E-42
APLF	x				AGAP004516	FBgn0026737	Not identified			tblastn to *An*. *gambiae* gene	
PNKP	x				AGAP012174	FBgn0037578	LLOJ000482	Scaffold1077: 23,640–25,668	Reverse	tblastn to *An*. *gambiae* gene	4.00E-24
APTX	x				AGAP004307	FBgn0038704	LLOJ001322	Scaffold126: 92,455–93,613	Forward	tblastn to *An*. *gambiae* gene	6.00E-29
Parp1		x			AGAP003230	FBgn0010247	LLOJ001283	Scaffold125: 56,700–70,597	Reverse	tblastn to *An*. *gambiae* gene	2.00E-60
Ligase 3		x			Absent	FBgn0038035	LLOJ008477	Scaffold71: 184,650–188,282	Reverse	tblastn to *D*. *melanogaster* gene	2.00E-43
Ligase 1		x			AGAP009222	FBgn0262619	LLOJ008476	Scaffold71: 183,315–187,114	Forward	tblastn to *An*. *gambiae* gene	0
Xrcc3		x			AGAP013180	FBgn0003480	LLOJ005967	Scaffold4: 274,950–281,740	Forward	tblastn to *An*. *gambiae* gene	2.00E-28
Xrcc1		x			AGAP002605	FBgn0026751	LLOJ004541	Scaffold268: 78,732–80,341	Forward	tblastn to *An*. *gambiae* gene	1.00E-47
ATM		x			AGAP009632	FBgn0045035	LLOJ002603	Scaffold17: 281,505–321,286	Reverse	tblastn to *An*. *gambiae* gene	1.00E-170
Mre11		x	x		AGAP006797	FBgn0020270	LLOJ006671	Scaffold485: 146,630–151,328	Forward	tblastn to *An*. *gambiae* gene	2.00E-157
Rad50		x	x		AGAP003676	FBgn0034728	LLOJ001007	Scaffold118: 147,175–151,533	Forward	tblastn to *An*. *gambiae* gene	0
Nbs1		x	x		AGAP003213	FBgn0261530	LLOJ000672	Scaffold1100: 41,240–43,475	Reverse	tblastn to *An*. *gambiae* gene	3.00E-54
Sae2		x	x		AGAP008637	FBgn0029113	LLOJ009358	Scaffold86: 50,707–65,797	Forward	tblastn to *An*. *gambiae* gene	0
Exo1			x		AGAP004491	FBgn0015553	LLOJ003541	Scaffold21: 238,272–240,297	Forward	tblastn to *An*. *gambiae* gene	7.00E-19
RPA			x		AGAP001421	FBgn0010173	LLOJ006328	Scaffold437: 68,232–71,334	Reverse	tblastn to *An*. *gambiae* gene	0
Sgs1			x		AGAP002967	FBgn0002906	LLOJ001099	Scaffold12: 322,054–329,185	Forward	tblastn to *An*. *gambiae* gene	0
Dna2			x		AGAP004685	FBgn0030170	LLOJ003812	Scaffold227: 12,526–19,819	Reverse	tblastn to *An*. *gambiae* gene	0
Rad51			x		AGAP013412	FBgn0003479	LLOJ000926	Scaffold1164: 24,172–26,802	Reverse	tblastn to *An*. *gambiae* gene	2.00E-171
Rad54			x		AGAP008748	FBgn0002989	LLOJ000406	Scaffold106: 72,542–75,384	Forward	tblastn to *An*. *gambiae* gene	0
BRCA2			x		AGAP007032	FBgn0050169	Not identified				
Polδ			x		AGAP011731	FBgn0263600	LLOJ000780	Scaffold113: 143,876–150,886	Forward	tblastn to *An*. *gambiae* gene	8.00E-26
Polθ			x		AGAP004615	FBgn0264326	Not identified				
Rad1				x	AGAP002255	FBgn0026778	LLOJ004989	Scaffold305: 92,142–93,895	Forward	tblastn to *An*. *gambiae* gene	1.00E-37
Rad10/Ercc1				x	AGAP004029	FBgn0028434	LLOJ005134	Scaffold320: 65,326–117,135	Forward	tblastn to *An*. *gambiae* gene	1.00E-16
Msh2				x	AGAP010282	FBgn0015546	LLOJ000903	Scaffold1156: 46,215–59,400	Reverse	tblastn to *An*. *gambiae* gene	0
Slx4				x	AGAP007582	FBgn0002909	Not identified				

A critical issue for genome engineering in sand flies is the size and the fragility of these insects. Screening of knock-outs requires extraction of gDNA. In mosquitoes, genotyping alive individuals can be performed with gDNA obtained from a single rear leg. In contrast, we found that sand flies are too fragile for this procedure. Excessive manipulation of alive individuals may result in stress and high mortality rates. Instead, genotyping can be performed after an individual has been already crossed, blood-fed and laid eggs using the live sand fly or its recently dead body as a source of DNA material using the Phire Animal Tissue Direct PCR Kit (Thermo Fisher Scientific). However, genotyping can be an issue if the identification of alive heterozygous individuals is needed for out-crossing to create a homozygous line. In this case, extracting gDNA from the pupal exuviae has been a valid option for other organisms [59]. It is important to emphasize the relevance of the proper design of the sgRNA. They should target genomic areas where no single nucleotide polymorphisms (SNPs) are present at inter and intra-individual level. Preliminary work of sequencing the target gene from several specimens is highly recommended before designing the sgRNA.

In this article, we described a protocol for sand fly embryo microinjection and addressed several issues related to microinjection and gene editing with a non-model organism. We believe gene editing in sand flies will provide essential information of great relevance to medicine and veterinary science on the biology of these vectors, and will further a better understanding of vector-parasite interactions.
